# FKBP5 Exacerbates Impairments in Cerebral Ischemic Stroke by Inducing Autophagy *via* the AKT/FOXO3 Pathway

**DOI:** 10.3389/fncel.2020.00193

**Published:** 2020-07-15

**Authors:** Shijia Yu, Mingjun Yu, Zhongqi Bu, Pingping He, Juan Feng

**Affiliations:** ^1^Department of Neurology, Shengjing Hospital of China Medical University, Shenyang, China; ^2^Department of Neurosurgery, Shengjing Hospital of China Medical University, Shenyang, China

**Keywords:** cerebral ischemic stroke, FKBP5, FOXO3, autophagy, ischemia and reperfusion injury

## Abstract

Cerebral ischemic stroke is regarded as one of the most serious diseases in the human central nervous system. The secondary ischemia and reperfusion (I/R) injury increased the difficulty of treatment. Moreover, the latent molecular regulating mechanism in I/R injury is still unclear. Based on our previous clinical study, we discovered that FK506 binding protein 5 (FKBP5) is significantly upregulated in patients, who suffered acute ischemic stroke (AIS), with high diagnostic value. Levels of FKBP5 were positively correlated with patients’ neurological impairments. Furthermore, a transient middle cerebral artery occlusion (tMCAO) model of mice was used to confirm that FKBP5 expression in plasma could reflect its relative level in brain tissue. Thus, we hypothesized that FKBP5 participated in the regulation of cerebral I/R injury. In order to explore the possible roles FKBP5 acted, the oxygen and glucose deprivation and reoxygenation (OGD/R) model was established to mimic I/R injury *in vitro*. FKBP5 expressing levels were changed by plasmid stable transfection. The altered expression of FKBP5 influenced cell viability and autophagy after OGD/R injury notably. Besides, AKT/FOXO3 cascade was involved in the FKBP5-regulating process. In the present study, FKBP5 was verified upregulated in cerebral I/R injury, related to the severity of ischemia and reperfusion injury. Additionally, our analyses revealed that FKBP5 regulates autophagy induced by OGD/R *via* the downstream AKT/FOXO3 signaling pathway. Our findings provide a novel biomarker for the early diagnosis of ischemic stroke and a potential strategy for treatment.

## Introduction

Cerebral ischemic stroke is a common neurological disease involving a loss of focal brain function, paralysis or numbness of the limbs, and aphasia (Zerna et al., 2018). There is a penumbra area around the ischemic core, where the cell injury could be reversible. Thrombolysis therapy is taken to save the penumbra so that the neurological impairments are attenuated. However, ischemia and reperfusion (I/R) injury in the penumbra area makes the treatment of cerebral ischemic stroke difficult (Powers et al., 2018).

Autophagy is a critical biological process for the metabolism and renewal of organelles. Autophagy-related cytoplasmic proteins or organelles can envelop or engulf substances to form vesicles, which combine with lysosomes to allow the degradation and metabolism of the vesicle components then (Kim et al., 2013). Under pathological conditions, autophagy is a process of dynamic changes, which can play either protective or destructive roles. Therefore, it is often regarded as a “double-edged sword.” It has been reported that autophagy is involved in the pathogenesis of ischemic stroke including apoptosis, oxidative stress, inflammation, and energy reduction (Wang et al., 2018). It was used to be considered that useless or harmful components could be cleared to protect cells during the formation of autophagosome. However, deuced autophagy could threaten lives due to excessive damages (Cuervo, 2004; Puyal and Clarke, 2009; Kim et al., 2013). Autophagy, which is induced by the I/R injury, can activate astrocytes to regulate normal functions of the brain through interacting with parenchymal cells (Dong et al., 2016; Han et al., 2018). Moreover, autophagic flux can regulate M1 phenotypic transformation of microglia in I/R injury-induced inflammatory response (Xia et al., 2016). In the previous study, we revealed that autophagy can aggravate cerebral I/R injury and decrease neuronal activity (Yu et al., 2019). However, the possible molecular mechanism still needs further exploration.

FK506 binding protein 5 (also called FKBP51) is a member of the immunoaffinity protein family and is involved in various biological processes, including immune regulation, protein folding, and transport (Sinars et al., 2003; Hahle et al., 2019; Hausl et al., 2019; Mao et al., 2019). As a co-chaperone in the glucocorticoid receptor (GR) complex, FKBP5 participates in the regulation of GR function along with heat shock protein 90 (HSP90; Binder, 2009). Increased FKBP5 has been detected for stress and glucocorticoid-stimulated conditions in the aging brain (Blair et al., 2013). Furthermore, FKBP5 is associated with heart attacks (Zannas et al., 2019). However, the precise changes in FKBP5 levels in cerebral ischemic stroke as well as the underlying mechanisms are still unclear.

In this study, we evaluated the relationship between FKBP5 expression in patient plasma samples and neurological impairments. Then, we explored pathways that were mediated by FKBP5 in I/R injury using *in vivo* and *in vitro* experiments. The results of this study improve our understanding of the latent pathological mechanisms in I/R injury and provide a new diagnostic biomarker.

## Materials and Methods

### Clinical Subjects

This study was exempt from approval requirements by the Institutional Review Board of Shengjing Hospital of China Medical University (IRB number, 2017PS161K). Fifty patients from January 2017 to March 2018 have participated in our study, who were in-patients in the Neurology Department of Shengjing Hospital in China Medical University and undertook the thrombolysis therapy within 3 h after the first onset of cerebral ischemic stroke. They were diagnosed based on the clinical manifestations, which were caused by focal neurological deficits. Brain scans, for example computed tomography (CT) or magnetic resonance imaging (MRI), were taken to support the diagnosis. Besides, two experienced neurologists judged the severity of neurological impairments using the National Institutes of Health Stroke Scale (NIHSS) after the thrombolytic therapy. Patients with severe systemic disease, cancer, trauma, and encephalorrhagia were excluded. Peripheral blood sample collection was conducted after the thrombolytic therapy. MRI with the diffusion-weighted imaging (DWI) sequences was recorded to evaluate the cerebral ischemia. The DWI volume of lesion was calculated by a semi-automated software (3D Slicer^1^). The control group was composed of 50 participants from the medical examination center in Shengjing Hospital for regular health checkup, without any history of stroke. All the subjects met the exclusion criteria.

### Focal Cerebral Ischemia in Mice

Institutional Animal Care and Use Committee of China Medical University approved all the animal experiments (IRB number, 2017PS035K). This study was conducted completely, complying with the National Institutes of Health Guide for the Care and Use of Laboratory Animals. The stress and pain of animals were minimized in our trial. We bought 50 male C57BL/6J mice with 22–25 g weight (8–10 weeks old) from Beijing HFK Bioscience Cooperation, China. Mice were fed in the 70% humidity environment at about 22°C following a 12-h day and night cycle, having free access to food and water. To establish the ischemia and reperfusion (I/R) injury model *in vivo*, a transient middle cerebral artery occlusion (tMCAO) operation was performed. The detailed procedures were described in our previous study (Yu et al., 2019). After anesthesia, the middle cerebral artery was occluded by a 6–0 silicone rubber-coated nylon monofilament (Beijing Cinontech Co. Ltd., Beijing, China), which was inserted *via* the external carotid artery (ECA) and internal carotid artery (ICA). The monofilament was removed 1 h later for reperfusion. During the whole procedure, the rectal temperature was kept at 37°C ± 0.5°C using a waterproof heat pad. Physiologic parameters (arterial blood pressure and heart rate) were measured at the tail artery using a smart noninvasive device (BP-98A; Softron, Tokyo, Japan). The regional cerebral blood flow (rCBF) related to the MCA was monitored by a laser Doppler flowmeter (FLO-C1; Omegaflo, Tokyo, Japan). It was considered as a successful modeling of MCAO with a reduction of more than 70% in rCBF (Chen et al., 2012).

### 2,3,5-Triphenyltetrazolium Chloride (TTC) Staining

The mouse brain tissue was stained with 2% 2,3,5-triphenyltetrazolium chloride (TTC, #T8877; Sigma, St. Louis, MO, USA) after a 24-hr reperfusion at 37°C. The infarct volume was assessed as we previously reported (Yu et al., 2019). A formula is listed as follows: [(volume of the contralateral hemisphere − volume of the nonischemic ipsilateral hemisphere)/volume of the contralateral hemisphere] × 100%.

### Enzyme-Linked Immunosorbent Assay

The protein expression of FKBP5 in human serum was detected using enzyme-linked immunosorbent assay (ELISA) kit (#SEE645Hu; Cloud-Clone Co., TX, USA). The relative concentrations were gained according to the standard curve through a microplate reader at 450 nm (BioTek, Winooski, VT, USA) according to a previous study (Chen and Gong, 2019).

### RNA Quantitative Detection

Total RNAs were isolated using TRIzol (#15596018; Life Technologies Corporation, Carlsbad, CA, USA). RNA concentrations and the quality were evaluated through a UV Spectrophotometer (N50 Touch; Implen, Germany). Real-time quantitative PCR was implemented using One-Step SYBR PrimeScript RT-PCR Kit (#RR064A; Takara Bio, Inc., Japan). Taking GAPDH as endogenous control, the relative expression of mRNA was calculated by 2^−ΔΔCt^ as previously reported (Yu et al., 2017). The primers were synthesized as follows: hsa-FKBP5: forward 5′-CAAGAAGTTTGCAGAGCAGGAT-3′ and reverse 5′-CACTGGGACTCTTCCCTCCTT-3′; mmu-FKBP5: forward 5′-GTACAACAAAGCCGTGGAGTG-3′ and reverse 5′-GCCCTGTTCTGAGGATTGACT-3′.

### Western Blot Assay

Brain tissue in the peri-infarct area was separated from the liquefied necrosis of the infarct core under the stereomicroscope, according to previous studies (Nagayama et al., 2000; Zhao et al., 2014). Total proteins were extracted from tissues and cells on the ice in a RIPA buffer with protease inhibitor (#R0278; Sigma, St. Louis, MO, USA). Proteins (60 μg each sample) were divided on an SDS–polyacrylamide gel electrophoresis (PAGE) through electrophoresis and transferred to polyvinylidene difluoride (PVDF) membranes (Millipore, Billerica, MA). Membranes were then blocked in 5% nonfat milk at room temperature for 2 h. Primary antibodies for FKBP5 (# 8245S; 1:1,000, Cell Signaling Technology, Beverly, MA, USA), LC3B (#3868; 1:1,000, Cell Signaling Technology, Beverly, MA, USA), SQSTM1 (#23214; 1:1,000, Cell Signaling Technology, Beverly, MA, USA), FOXO3 (#2497; 1:1,000, Cell Signaling Technology, Beverly, MA, USA), p-FOXO3 (#9466; 1:1,000, Cell Signaling Technology, Beverly, MA, USA), AKT (#4691; 1:1,000, Cell Signaling Technology, Beverly, MA, USA), p-AKT (#4060; 1:2,000, Cell Signaling Technology, Beverly, MA, USA), and GAPDH (#sc-365062; 1:1,000, Santa Cruz Biotechnology, CA, USA) were incubated at 4°C overnight. Then, HRP-conjugated secondary antibodies (#SA00001-1 or # SA00001-2; 1:10,000, Proteintech, Chicago, IL, USA) were incubated at room temperature for 2 h. Target bands were visualized under a chemiluminescence imaging analysis system (Amersham Imager 600, GE, CT, USA) by an enhanced chemiluminescence detection kit (ECL kit, # WBKLS0500; Millipore, Billerica, MA). ImageJ software was applied to calculate the integrated density values. Moreover, the relative integrated density values were normalized to GAPDH.

### Cell Culture, Transfection, and Treatment

Neuro-2a cells were cultured using medium containing 10% (v/v) fetal bovine serum (FBS; Gibco, Carlsbad, CA, USA) in Dulbecco’s modified Eagle medium (DMEM)/high glucose, according to our early research (Yu et al., 2019). Cells were preplanted into 24-well plates. After cultured to ~70% confluency, Neuro-2a cells were transfected with plasmids of FKBP5(+) for overexpression and FKBP5(−) for knockdown or their relevant noncoding (NC) sequences, which were constructed by GenePharma Corporation (Shanghai, China). Opti-MEM and Lipofectamine 3,000 reagents (#L3000015; Invitrogen, CA, USA) were applied according to the manufacturer’s instructions. Stable transfected cell lines were obtained by growing a gradient of G418 (#G5013; Sigma-Aldrich, St. Louis, MO, USA) density. The oxygen-glucose deprivation for 6 h and reoxygenation for 24 h (OGD/R) cell model was used to explore the I/R injury mechanism *in vitro* as before Yu et al. (2019). LY294002 (#L9908; 10 μM, Sigma-Aldrich, St. Louis, MO, USA) was taken as an AKT inhibitor. The plasmid sequences were listed as followed: FKBP5(+): 5′-ATGACTACTGATGAGGGCACC-3′; FKBP5(−): 5′-GCGTTATCCGTAGAATCAAAC-3′.

### Cell Viability Determination

Cell Counting Kit 8 (#CK04-01; CCK8, Dojindo, Japan) assay was used to determine Neuro-2a cell viabilities. After being seeded into 96-well plates overnight, cells were treated with OGD/R. 10 μl CCK8 solution was added to the culture medium per well at 37°C 24 h later. The cell viabilities were detected by a microplate reader (BioTek, Winooski, VT, USA). The results were normalized to the control group and expressed as the percentage.

### Immunofluorescence Staining

Cells were fixed in cold methanol at −20°C. After blocking in 5% normal goat serum, cells were incubated with the primary antibody against LC3B (#3868; 1:100, Cell Signaling Technology, Beverly, MA, USA) at 4°C overnight and then TRITC-conjugated anti-rabbit IgG (#SA00007-2; 1:100, Proteintech, Chicago, IL, USA) for 2 h at room temperature (Li et al., 2017). Nuclei were stained by 4′,6-diamidino-2-phenylindole (DAPI, # D9542; Sigma Aldrich, St. Louis, MO, USA). Images of cells were gained *via* a laser-scanning confocal microscope (Nikon, Japan).

### Transmission Electron Microscopy

Cells were gathered in EP tubes using 0.1 M phosphate buffer (pH 7.4) containing 2.5% glutaraldehyde to prefix for more than 2 h. After being washed in PBS for three times, cells were postfixed in 0.1 M phosphate buffer (pH 7.4) with 1% osmium tetroxide for 30 min. Then, the samples were dehydrated by acetone with different gradient. Pure acetone and embedding agents were used to infiltrate and embed. Samples were sectioned to 70–80 nm and double stained with 3% uranyl acetate–lead citrate for the transmission electron microscopy (TEM, Hitachi, Japan) observation (Harris, 2015; Wu et al., 2019).

### Autophagy Flux Assessment

Adenovirus labeling mRFP-GFP-LC3 (Han Bio, Shanghai, China) was used to infect cells. The medium was replaced after 48 h of infection. Then, OGD/R treatment was undertaken to treat the infected cells. After reoxygenating for 24 h, we removed the medium and washed cells with PBS for three times. Then, 4% paraformaldehyde was applied to fix cells for 30 min (Zhang et al., 2016). After PBS washing for another three times, DAPI (# D9542; Sigma Aldrich, St. Louis, MO, USA) was used to display the nuclei (Zhang et al., 2016). Fluorescence was recorded under a two-photon microscope (Zeiss, Germany).

### Statistical Analyses

All data were statistically analyzed using IBM SPSS statistics 23.0 (IBM Corp., Armonk, NY, USA). Continuous variables were expressed as mean ± SD. Categorical variables were expressed as counts, and proportions were obtained after the patients’ total number divided by the number of events. Experiments were repeated for three times. Univariate analyses were proceeded *via* the Student *t*-test, ANOVA test, Mann–Whitney U test, or *χ*^2^ test. The association between FKBP5 and the severity of neurological impairments or lesion volume was evaluated by Spearman correlation analysis. *P* < 0.05 was regarded as statistically significant.

## Results

### FKBP5 Is Upregulated in Patients With Ischemic Stroke

**Figure 1:**
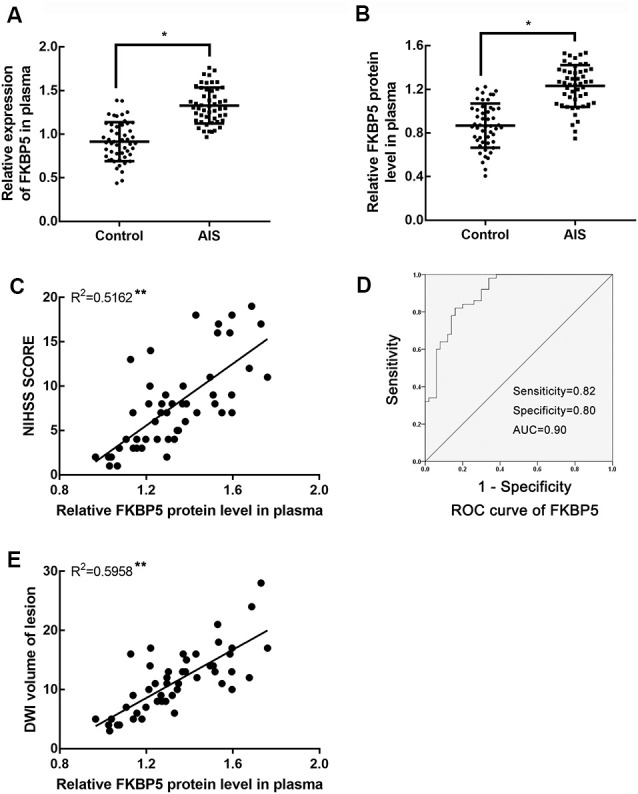
FK506 binding protein 5 (FKBP5) was upregulated notably in acute ischemic stroke (AIS) patients. **(A)** Expression of FKBP5 in plasma of AIS patients (*n* = 50) and healthy controls (*n* = 50) examined by real-time qPCR. **(B)** Expression of FKBP5 in plasma of AIS patients (*n* = 50) and healthy controls (*n* = 50) tested by ELISA. Data are presented as the mean ± SD. *P < 0.05 vs. health control group. **(C)** Linear regression analysis was applied in each individual between the FKBP5 expression and National Institute of Health Stroke Scale (NIHSS) score, **P < 0.01. **(D)** Receiver-operator characteristic (ROC) curve of FKBP5 in the diagnosis of AIS (*n* = 50). **(E)** Linear regression analysis of the relationship between FKBP5 expression and diffusion-weighted imaging (DWI) volume of lesion, **P < 0.01.

To determine whether FKBP5 is involved in ischemic stroke, we examined its expression in patients with acute ischemic stroke (AIS). In particular, we quantitatively evaluated FKBP5 levels in plasma samples obtained from AIS patients who had received thrombolytic therapy (*n* = 50) and healthy participants (*n* = 50). A comparison of basic characteristics between the two groups is provided in Table 1. Some of the acknowledged risk factors of stroke, including current smoking, alcohol consumption, hypertension, diabetes, and carotid stenosis, were taken into consideration. Drinking alcohol consecutively for more than 3 months before stroke was considered as alcohol consumption. DWI-ASPECTS were evaluated by two radiologists according to previous studies (Nezu et al., 2010; Lassalle et al., 2016). Both quantitative real-time PCR (qRT-PCR) and enzyme-linked immunosorbent assays (ELISA) indicated that plasma FKBP5 expression is higher in AIS patients than healthy individuals (Figures 1A,B). Furthermore, the level of FKBP5 increased with the growing National Institute of Health Stroke Scale (NIHSS) score (*R*^2^ = 0.5162, *P* < 0.05; Figure 1C). Also, it had a positive correlation with DWI volume of lesion (*R*^2^ = 0.5958, *P* < 0.05; Supplementary Figure S1). A ROC curve was used to assess the predictive value of FKBP5 for the diagnosis of ischemic stroke with sensitivity of 0.82 and specificity of 0.80. The area under the ROC curve (AUC) is 0.90 (Figure 1D). As the relative FKBP5 expression rising, the DWI volume of lesion in patients expands (*R*^2^ = 0.5958, *P* < 0.05; Figure 1E).

**Table 1:** Baseline characteristics of the subjects.

	AIS, *n* = 50	Control, *n* = 50	*P*
Age	63.76 ± 8.487	62.50 ± 7.313	0.428
Male (%)	27 (54.0)	23 (46.0)	0.689
BMI (kg/m^2^)	26.9 ± 4.98	25.4 ± 5.61	0.161
Waist circumference (cm)	92.4 ± 9.21	88.9 ± 11.33	0.093
Risk factors, *n* (%)			
Current smoking	22 (44.0)	16 (32.0)	0.216
Alcohol consumption	12 (24.0)	8 (16.0)	0.317
Hypertension	36 (72.0)	13 (26.0)	<0.001
Diabetes	23 (46.0)	19 (38.0)	0.418
Carotid stenosis	14 (28.0)	10 (20.0)	0.349
Laboratory tests			
Total cholesterol (mmol/L)	4.57 ± 1.12	4.64 ± 0.80	0.086
Triglyceride (mmol/L)	1.52 ± 0.82	1.46 ± 0.85	0.993
LDL-C (mmol/L)	2.93 ± 0.90	2.95 ± 0.73	0.406
HDL-C (mmol/L)	1.11 ± 0.37	1.23 ± 0.27	0.264
NIHSS score, n (%)			
1–4	17 (34.0)		
5–15	26 (52.0)		
16–25	7 (14.0)		
Imaging data			
DWI volume of lesion (cm^3^)	11.18 ± 5.43		
DWI-ASPECTS, n (%)			
0–4	5 (10.0)		
5–7	24 (48.0)		
8–10	21 (42.0)		
Vessel occlusion			
Any	14 (26.0)		
Internal carotid artery	2 (4.0)		
Middle cerebral artery (M1)	13 (26.0)		
Middle cerebral artery (M2)	19 (38.0)		
Posterior cerebral artery	1 (2.0)		
Basilar artery	1 (2.0)		

*DWI-ASPECTS: scoring of Alberta Stroke Program Early CT Score using DWI*.

### FKBP5 Is Upregulated in Transient Middle Cerebral Artery Occlusion (tMCAO)

**Figure 2:**
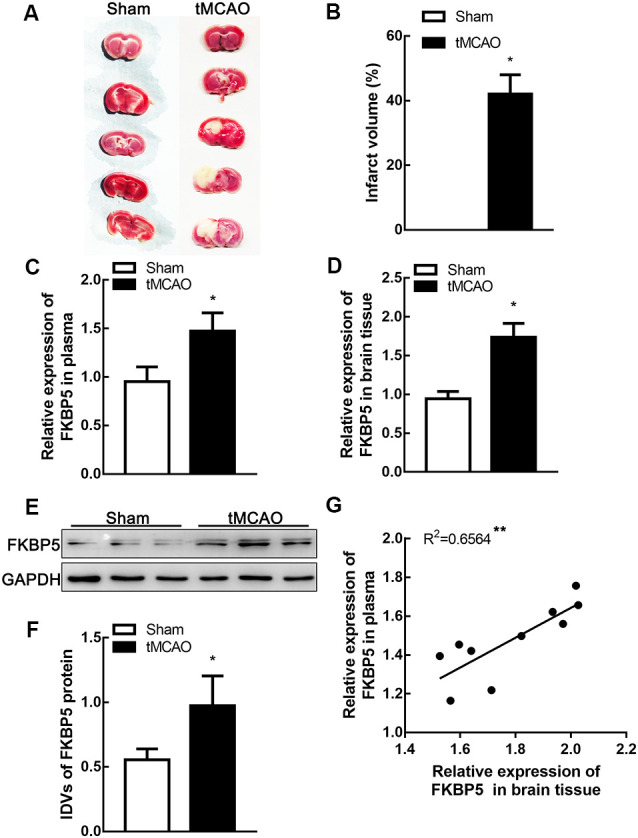
FKBP5 was significantly upregulated in transient middle cerebral artery occlusion (tMCAO) mice. **(A,B)** Infarct volume in the brain tissues of tMCAO and sham mice (*n* = 6 each group). **(C,D)** Expression of FKBP5 in plasma and brain tissue of tMCAO and sham mice detected by real-time qPCR (*n* = 10 each group). **(E,F)** Expression of FKBP5 in the brain tissue of tMCAO and sham mice detected by Western blot assay (*n* = 10 each group). IDV is short for integrated density values. Data are presented as the mean ± SD. *P < 0.05 vs. sham group. **(G)** Linear regression analysis was performed in each tMCAO mouse between the FKBP5 expression in brain tissue and plasma (*n* = 10 each group), **P < 0.01.

To explore the role of FKBP5 in ischemic brain tissues, mice were treated by tMCAO and their brain tissues were separated for further assays. The infarct volume in tMCAO mouse brains was about 40% (Figures 2A,B). Relative levels of FKBP5 mRNA in both plasma and brain tissues were much higher in the tMCAO group (Figures 2C,D). Based on Western blotting, FKBP5 protein levels were markedly increased in the tMCAO group, as shown in Figures 2E,F. Furthermore, there was a positive correlation between the FKBP5 levels in the plasma and brain lesion of mice subjected to tMCAO (*R*^2^ = 0.6564, *P* < 0.01; Figure 2G).

### OGD/R Treatment Improves mRNA and Protein Levels of FKBP5

**Figure 3:**
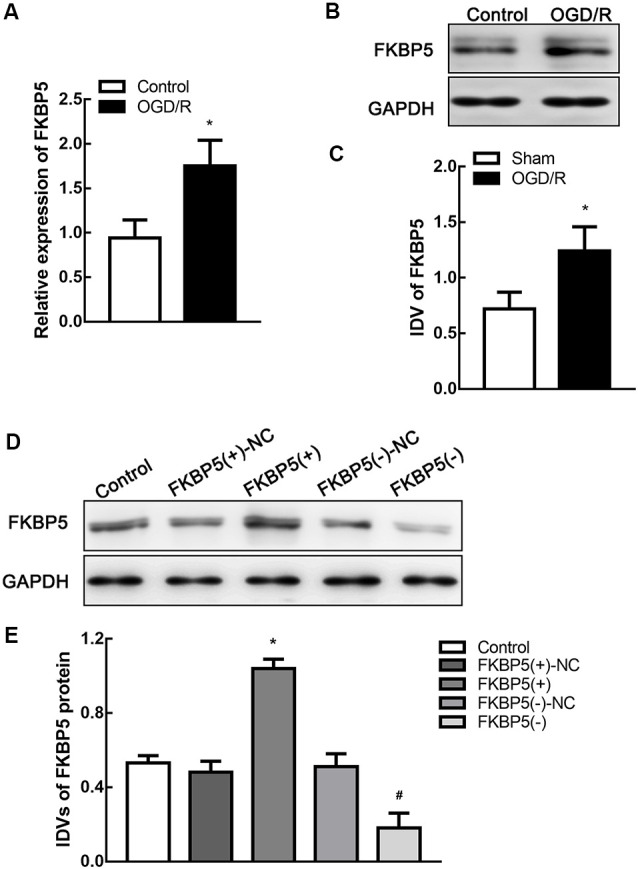
FKBP5 was upregulated in oxygen and glucose deprivation and reoxygenation (OGD/R) cells and establishments of stable transfected cell lines. **(A)** Expression of FKBP5 in OGD/R and control group quantified by real-time qPCR. **(B,C)** Expression of FKBP5 in OGD/R and control group determined by Western blot assay. *P < 0.05 vs. control group. **(D,E)** Protein levels of FKBP5 after stably transfecting plasmids with FKBP5(+), FKBP5(−), and their relative noncoding (NC) sequences. Data are presented as the mean ± SD. *P < 0.05 vs. FKBP5(+)-NC group. ^#^*P* < 0.05 vs. FKBP5(−)-NC group. For **(A–E)**, *n* = 3 in each group.

Cells were treated by oxygen and glucose deprivation and reoxygenation (OGD/R) to mimic I/R injury *in vitro*. After OGD/R treatment, both mRNA and protein levels of FKBP5 improved significantly (*P* < 0.05; Figures 3A–C), which were consistent with those in tMCAO mouse brain. To determine the mechanism underlying the role of FKBP5 in I/R injury, stably transfected Neuro-2a cell lines were established to either overexpress or silence FKBP5. The transfective efficiency is shown in Figures 3D,E.

### FKBP5 Is Related to Cell Viability and Autophagy in OGD/R

**Figure 4:**
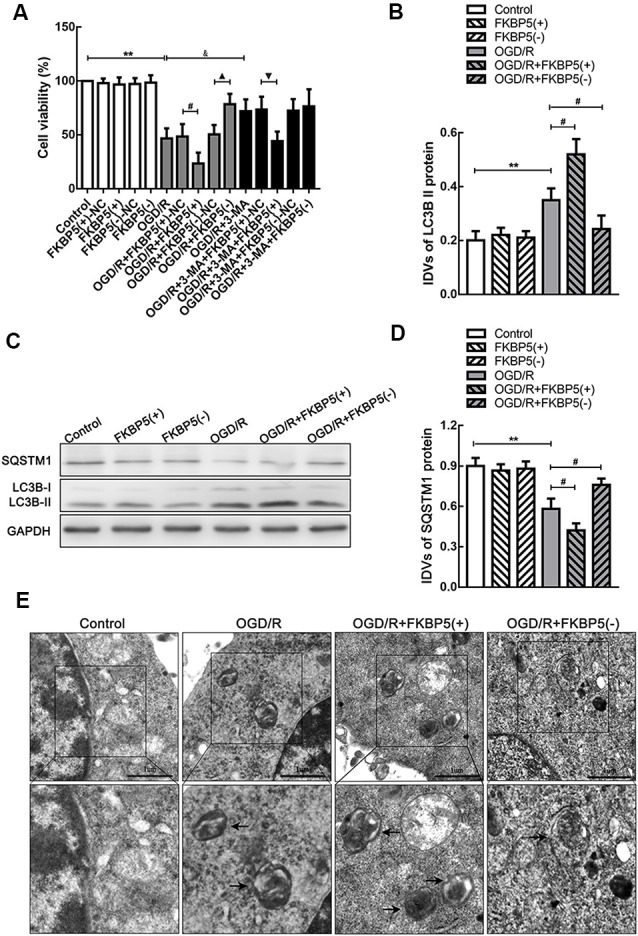
FKBP5 aggravated cell damage *via* autophagy. **(A)** CCK-8 assay was performed to assess the influences of FKBP5 and autophagy on cell viability. **P < 0.01 vs. control group. ^#^*P* < 0.05 vs. OGD/R + FKBP5(+)-NC group. ^▴^*P* < 0.05 vs. OGD/R + FKBP5(−)-NC group. ^&^*P* < 0.05 vs. OGD/R group. ^▾^*P* < 0.05 vs. OGD/R + 3-MA+FKBP5(+)-NC group. **(B–D)** Western blot analysis of SQSTM1 and LC3B II expression in FKBP5 stably transfected cell lines. GAPDH was regarded as an endogenous control. Data are presented as the mean ± SD. Statistical analysis was conducted with nonparametric Mann–Whitney test. **P < 0.01 vs. control group. ^#^*P* < 0.05 vs. OGD/R group. **(E)** TEM was used to observe the autophagosomes in OGD/R cells with altered FKBP5 expression. Arrows point to autophagic vacuoles (AVs) with double membranes. Scale bars represent 1 μm. For **(A–E)**, *n* = 3 in each group.

To determine the effects of FKBP5 in OGD/R, we evaluated Neuro-2a cell viability and autophagy. OGD/R treatment could impair cell viability. Moreover, the overexpression of FKBP5 could aggravate the impairment of cell viability, while knockdown of FKBP5 could alleviate it (Figure 4A). Since LC3B and SQSTM1 are important factors in the process of autophagy, we tested their expressions by Western blot assay. It was found that LC3B II levels increased substantially in the FKBP5-overexpressing group after OGD/R treatment (Figures 4B,C), while SQSTM1 levels decreased conversely (Figures 4B,D). Then, transmission electron microscopy was used to observe autophagic vacuoles in cells directly. After OGD/R treatment, more autophagic vacuoles appeared in cells, indicating an activation of autophagy. Furthermore, the number of autophagic vacuoles was elevated much more with FKBP5 overexpression (Figure 4E).

**Figure 5:**
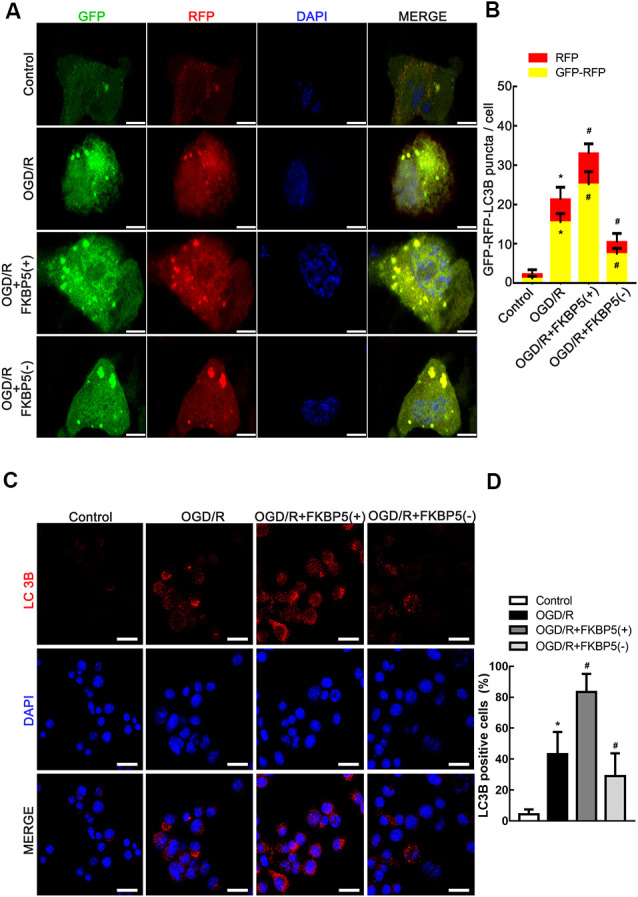
FKBP5-mediated autophagy induced by OGD/R. **(A)** The mRFP-GFP-LC3B adenovirus was infectious for autophagy flux to be monitored in OGD/R cells with FKBP5 expression alteration. Scale bars represent 10 μm. **(B)** During each experiment, more than 100 cells were counted. Data are presented as the mean ± SD. **(C)** Immunofluorescence staining assay with red dots for LC3B expression in the OGD/R group changing with FKBP5 expression. Scale bars represent 40 μm. **(D)** More than 100 cells were taken into account. Experiments were repeated three times. *P < 0.05 vs. control group. ^#^*P* < 0.05 vs. OGD/R group. For **(A–D)**, *n* = 3 in each group.

LC3B was fluorescently tagged by ad-mRFP-GFP-LC3B, which was infected into Neuro-2a cells to trace autophagic flux. Green fluorescent protein is unstable in acidic environments. Therefore, in an environment of pH <5, such as autolysosomes, only red fluorescence was displayed. Thus, early autophagosomes could exhibit yellow fluorescence as a result of the overlapping red and green signals. The yellow and red fluorescence signals were observed extensive and strong in the OGD/R group, while they turned less and weaker after FKBP5 knockdown (Figures 5A,B). An immunofluorescence analysis was performed to monitor the alteration in the distribution of LC3B, which was an autophagosome marker. Bright and punctate dots representing LC3B were detected in cells overexpressing FKBP5 after being subjected to OGD/R injury (Figures 5C,D).

### FKBP5-Mediated Autophagy *via* the AKT/FOXO3 Pathway in OGD/R

**Figure 6:**
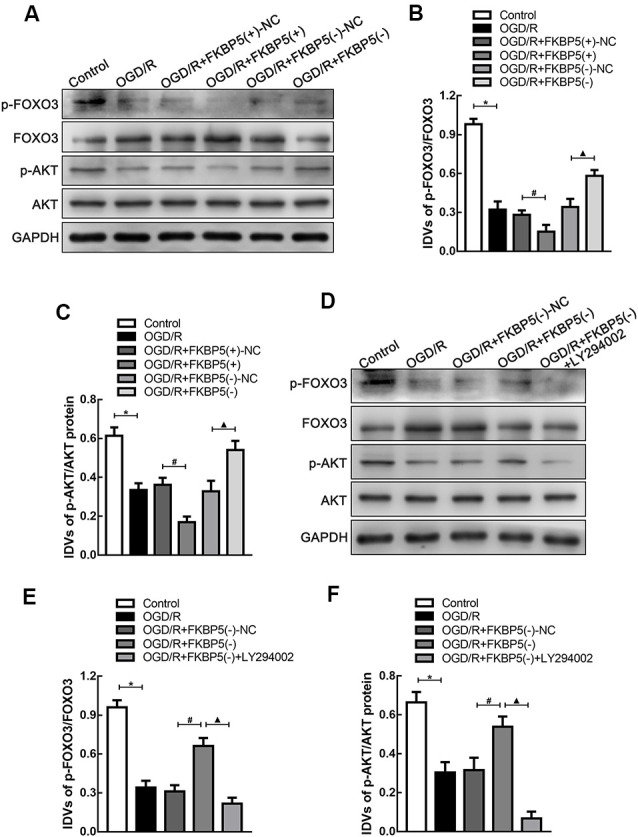
FKBP5-regulated AKT/FOXO3 signaling pathway. **(A–C)** Protein levels of p-FOXO3/FOXO3 and p-AKT/AKT in the OGD/R group with altered FKBP5 expression. *P < 0.05 vs. control group. ^#^*P* < 0.05 vs. OGD/R + FKBP5(+)-NC group. ^▴^*P* < 0.05 vs. OGD/R + FKBP5(−)-NC group. **(D–F)** Western blot analysis of p-FOXO3, FOXO3, p-AKT, and AKT expression in OGD/R cells with the treatment of FKBP5(−) and LY294002. GAPDH was regarded as an endogenous control. *P < 0.05 vs. control group. ^#^*P* < 0.05 vs. OGD/R + FKBP5(−)-NC group. ^▴^*P* < 0.05 vs. OGD/R + FKBP5(−) group. For **(A–F)**, *n* = 3 in each group.

To explore the regulating mechanism of FKBP5 in OGD/R injury, the AKT/FOXO3 cascade was evaluated owing to its crucial role in autophagy. As shown in Figures 6A–C, rapid declines in p-FOXO3 and p-AKT expression were detected in the OGD/R group, and they were decreased more obviously in the FKBP5-overexpressing group. These results indicated that the AKT/FOXO3 axis is inhibited by high levels of FKBP5. Then, LY294002 was used to specifically inhibit the AKT/FOXO3 axis. As shown in Figures 6D–F, the expression levels of p-AKT and p-FOXO3 in cells with FKBP5 knockdown were notably increased. However, the changes in p-AKT and p-FOXO3 were rescued by LY294002, suggesting that FKBP5 regulates OGD/R-induced autophagy *via* the AKT/FOXO3-dependent pathway.

## Discussion

In our study, we detected increasing mRNA and protein levels of FKBP5 in AIS patients’ plasma. Moreover, our results implied that FKBP5 has a high diagnostic value of cerebral ischemic stroke. Furthermore, using the tMCAO model to study *in vivo*, we determined that after cerebral ischemia, the relative expression of FKBP5 in the plasma might reflect it in brain tissues. The OGD/R model was taken to develop the underlying molecular mechanism. Overexpression of FKBP5 could activate autophagy and decrease cell viability after OGD/R injury. The possible regulating role of FKBP5 in OGD/R injury may act *via* the AKT/FOXO3 pathway. As the most common disease of the nervous system in the elderly, cerebral ischemic stroke threatens the life of nearly 10 million people every year (Writing Group Members et al., 2016). Thrombolytic therapies, including the use of tissue plasminogen activator (tPA) as the only FDA-approved drug, have shown unsatisfied efficacies in some way. Hence, an effective diagnostic marker is needed to direct new treatment strategies (Tuttolomondo et al., 2016).

Here, we found that FKBP5 is involved in the evolution of acute cerebral ischemic stroke. FKBP5 levels increased in the AIS patients’ plasma after thrombolysis treatments. A higher expression of FKBP5 was tested in the plasma from patients who suffered larger cerebral infarction. Additionally, the diagnostic value of FKBP5 was high. We observed a correlation between the expression of FKBP5 in the plasma and brain tissue of tMCAO mice, suggesting that levels of FKBP5 in the peripheral blood could reflect it in the ischemic brain to some extent.

Autophagy is a conserved process in eukaryotes with the main character of “self-eating” (Tai et al., 2017). Basal autophagy is essential for maintenance of homoeostasis, because cells are especially vulnerable to the growing defective proteins and organelles during the post-mitotic phase. Dysfunctional autophagy could break down the internal balance of multiple metabolic processes. Reports indicate that dysfunctional autophagy is associated with such diseases as neurodegeneration, carcinoma, and heart attack (Lumkwana et al., 2017; Ma et al., 2018; Hu et al., 2019). However, the role that autophagy plays in cerebral ischemic stroke remains controversial. Evidences show that inhibition of autophagy can reduce amyloid β deposit and prevent secondary neurodegenerative damage following focal cerebral infarction in mammalian (Xing et al., 2012). Some researches indicate that brain injury after I/R may be aggravated because induced autophagy can cause neuron apoptosis *via* a Beclin1-independent pathway (Grishchuk et al., 2011; Han et al., 2018). Autophagy can increase the blood–brain barrier (BBB) permeability by mediating ZO-1 redistribution in the early reperfusion of stroke (Zhang et al., 2018). Furthermore, activated autophagy in ischemia contributes to astrocytes death *via* regulating the cathepsin-related mitochondrial pathway (Zhou et al., 2017b), while others reported that inhibiting autophagy flux could induce the M1 phenotype of microglial cells and raise tumor necrosis factor alpha (TNF-α) expression, to promote inflammatory reflection (Xia et al., 2016). Moreover, it also suggested that mild hypothermia could protect neurons against OGD/R *via* enhancing autophagic flux (Zhou et al., 2017a). This may be due to that autophagy itself is a dynamic process, which can protect cells from dysfunction at the early stage, but harmful over time. In our previous study, it was proven that activated autophagy could reduce cell viability by promoting neuronal apoptosis in I/R injury with a 24-h reperfusion (Yu et al., 2019). We further studied the possible regulating mechanisms in this research.

FKBP5 is implicated in stress responses, inflammation, and the risk of various diseases (Maiaru et al., 2016). For example, FKBP5 is upregulated in elderly patients with acute myocardial infarction (Zannas et al., 2019). Additionally, there is evidence that FKBP5 may interact with inflammatory factors in cardiovascular diseases (Vaccarino et al., 2013). Increasing FKBP5 could promote the NF-κB regulation of kinases and inflammation (Yehuda et al., 2016). In malignant melanoma subjected to ionizing radiation, FKBP5 was related to apoptosis resistance while inducing autophagy (Romano et al., 2010). During antidepressant treatments, it was considered that FKBP5 might be a potential target of some drugs related with autophagy (Gassen et al., 2014). However, there are few studies that focus on FKBP5 regulating autophagy and apoptosis in cerebral I/R injury. Our study revealed a decrease in cell viability when FKBP5 was overexpressed, implying that high levels of FKBP5 might aggravate neuronal injury.

Since the activation of autophagy could aggravate OGD/R injury, we hypothesized that FKBP5 influences cell viability by promoting autophagy. We assessed autophagy in OGD/R-treated cells with an altered expression of FKBP5 by Western blotting, immunofluorescence, and transmission electron microscopy. Since LC3BII and SQSTM1 are two vital proteins involved in autophagy, the increase in LC3BII and decrease in SQSTM1 observed in our study indicated that FKBP5 overexpression could induce autophagy after OGD/R treatment. Conversely, low FKBP5 expression might suppress autophagy in OGD/R. Furthermore, the expression and distribution of LC3B were changed according to immunofluorescent staining with different FKBP5 expressions. By transmission electron microscopy, we detected a strong increase in the number of autophagosomes in FKBP5-overexpressing cells, which were subjected to OGD/R treatment. After ad-mRFP-GFP-LC3B infection, autophagic flux increased with high FKBP5 expression, while it decreased in response to FKBP5 knockdown. These results implied that FKBP5 might participate in the regulation of autophagy by affecting autophagic flux (Klionsky et al., 2016). However, further studies are needed to clarify the specific roles of FKBP5 in autophagic flux.

AKT is regarded as an important molecule in regulating autophagy. Studies showed that AKT is associated with the regulation of autophagy in diabetes mellitus (Jahng et al., 2019). It was revealed that AKT could mediate Beclin 1 phosphorylation to form a filament complex as an autophagy inhibitory in human cancer (Wang et al., 2012). Other researchers found that kinase mammalian target of rapamycin (mTOR) as a critical molecule in autophagy reaction was regulated by AKT as well. The AKT/mTOR pathway was reported to reduce inflammatory response by modulating autophagy in osteoarthritis (Xue et al., 2017). The clearance of defective proteins in neurodegenerative diseases was also altered (Heras-Sandoval et al., 2014). Except for these molecules, FOXO3 is considered as another important downstream target of the PI3K/AKT cascade (Eijkelenboom and Burgering, 2013). FOXO3, which is a member of the Forkhead box O (FOXO) transcription factor family, plays a crucial role in deciding the cell’s fate. Activated AKT can phosphorylate FOXO3 and modulate its functions (Guo and Sonenshein, 2004). Evidence showed that the AKT/FOXO3 pathway participates in the regulation of cell proliferation and apoptosis in carcinoma (Cao et al., 2018; Yue and Sun, 2018). As reported, the AKT/FOXO3 pathway is also associated with the regulation of mitochondrial apoptosis (Pagano et al., 2019). Previous evidence elucidated that FKBP5 impacted autophagy markers Beclin1 and LC3B, relying on AKT phosphorylation (Gassen et al., 2014). Besides, FKBP5 could promote the interaction between AKT and PHLPP as a scaffolding protein and influence the phosphorylation of downstream FOXO3 as a tumor suppressor (Pei et al., 2009). Based on the abovementioned findings, we wondered whether FKBP5 could also affect autophagy induced by brain I/R injury *via* regulating the AKT/FOXO3 cascade. In our study, we discovered that the AKT/FOXO3 pathway had a neuroprotective effect when it was activated. These findings were in accordance with those of previous studies (She et al., 2018). Our analyses also showed that FKBP5 mediates OGD/R-induced autophagy *via* the AKT/FOXO3-dependent signaling pathway. However, there may be other molecular mechanisms involved in the FKBP5 regulation of autophagy, and it still needs further studies for more information.

In conclusion, our findings demonstrated that FKBP5 expression increased in ischemic stroke patients for the first time and contributed to the latent regulatory mechanism of I/R injury. The overexpression of FKBP5 could inhibit downstream AKT and FOXO3 phosphorylation, thereby inducing autophagy and leading to reduction in cell viability. Our results provide FKBP5 as a novel biological marker for diagnosis and a potential treatment strategy for cerebral ischemic stroke. However, there are some limitations in our study. Follow-up studies of clinical subgroups will be carried out for further study, to explore the specific roles of FKBP5 in the development of cerebral ischemic stroke. Besides, more studies need to be put into effect about the latent influence of thrombolytic therapy on FKBP5 expression. Other possible regulating mechanisms will be explored as well.

## Data Availability Statement

The raw data supporting the conclusions of this article will be made available by the authors, without undue reservation.

## Ethics Statement

Ethical review and approval was not required for the study on human participants in accordance with the local legislation and institutional requirements. Written informed consent (from the patients/participants or patients/participant’s legal guardian/next of kin) was not required to participate in this study in accordance with the national legislation and the institutional requirements.

## Author Contributions

SY designed and performed the experiments, analyzed the data, and wrote the manuscript. MY performed the bioinformatics analysis. ZB and PH performed the statistical analyses. JF revised the manuscript. All authors approved the final version of the manuscript.

## Conflict of Interest

The authors declare that the research was conducted in the absence of any commercial or financial relationships that could be construed as a potential conflict of interest.

## Footnotes

^1^https://www.slicer.org, (Mechtouff et al., 2019; Wiedemann et al., 2019).

## Supplementary Material

FIGURE S1 **(A)** Linear regression analysis was performed in each patient between the FKBP5 expression and DWI volume of lesion, ***P* < 0.01. **(B)** Quantification of autophagic vacuoles (AVs). More than 40 cells were counted in each experiment. ns, not significant, **P* < 0.05 vs. control group. ^#^*P* < 0.05 vs. OGD/R group. For **(B,C)** data are presented as the mean ± SD (*n* = 3 in each group). **(C)** Linear regression analysis was performed in each mouse between the FKBP5 expression and cerebral infarct volume.

FIGURE S2 Uncropped blot images.
