# Supplementary figures and images for: FKBP5 Exacerbates Impairments in Cerebral Ischemic Stroke by Inducing Autophagy via the AKT/FOXO3 Pathway
Source: Front Cell Neurosci. 2020 Jul 15;14:193. doi: 10.3389/fncel.2020.00193 (PMC7374263; doi:10.3389/fncel.2020.00193)

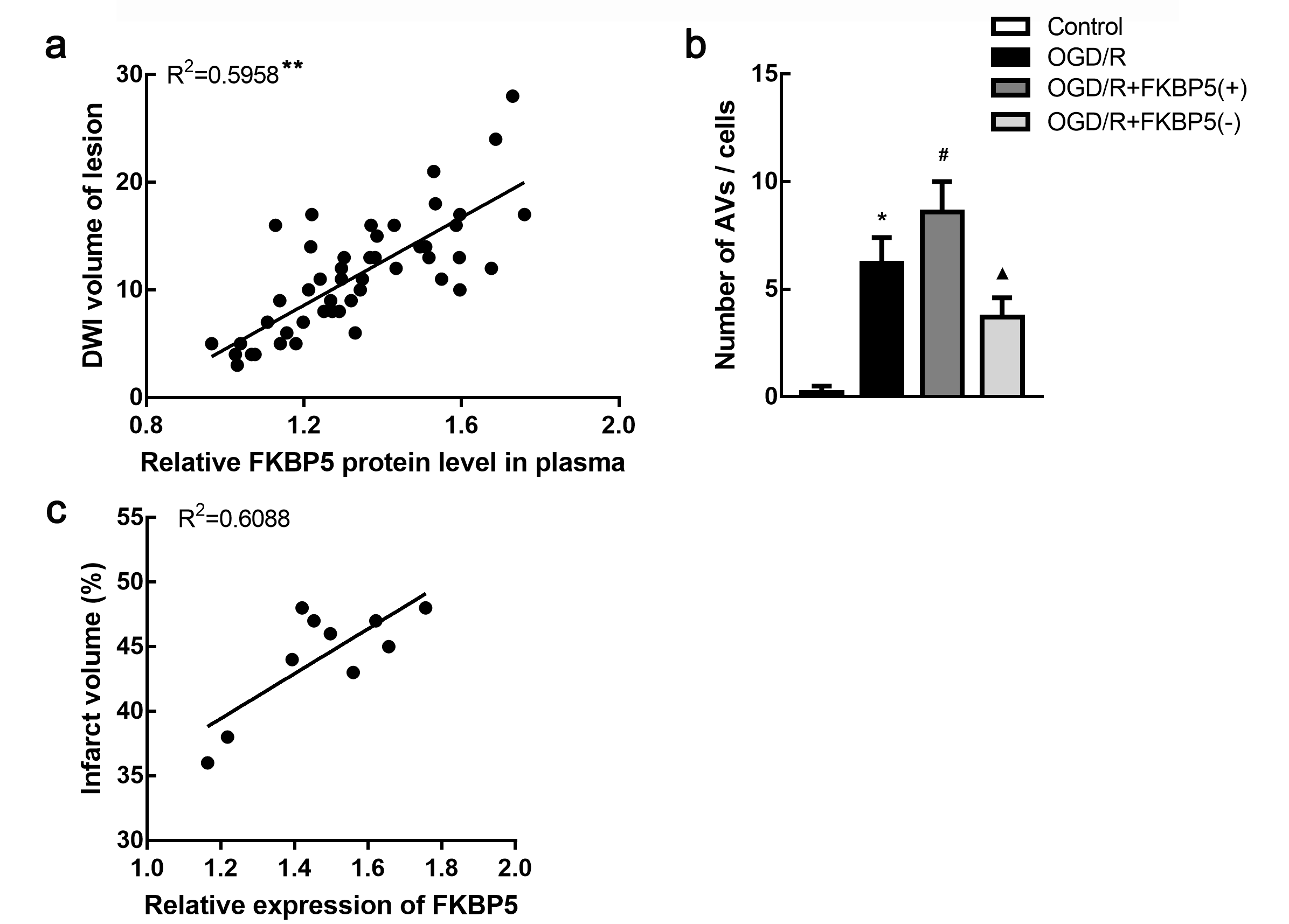

Supplement: FIGURE S1 — (A) Linear regression analysis was performed in each patient between the FKBP5 expression and DWI volume of lesion, **P < 0.01. (B) Quantification of autophagic vacuoles (AVs). More than 40 cells were counted in each experiment. ns, not significant, *P < 0.05 vs. control group. #P < 0.05 vs. OGD/R group. For (B,C) data are presented as the mean ± SD (n = 3 in each group). (C) Linear regression analysis was performed in each mouse between the FKBP5 expression and cerebral infarct volume. [file Image_1.TIF]

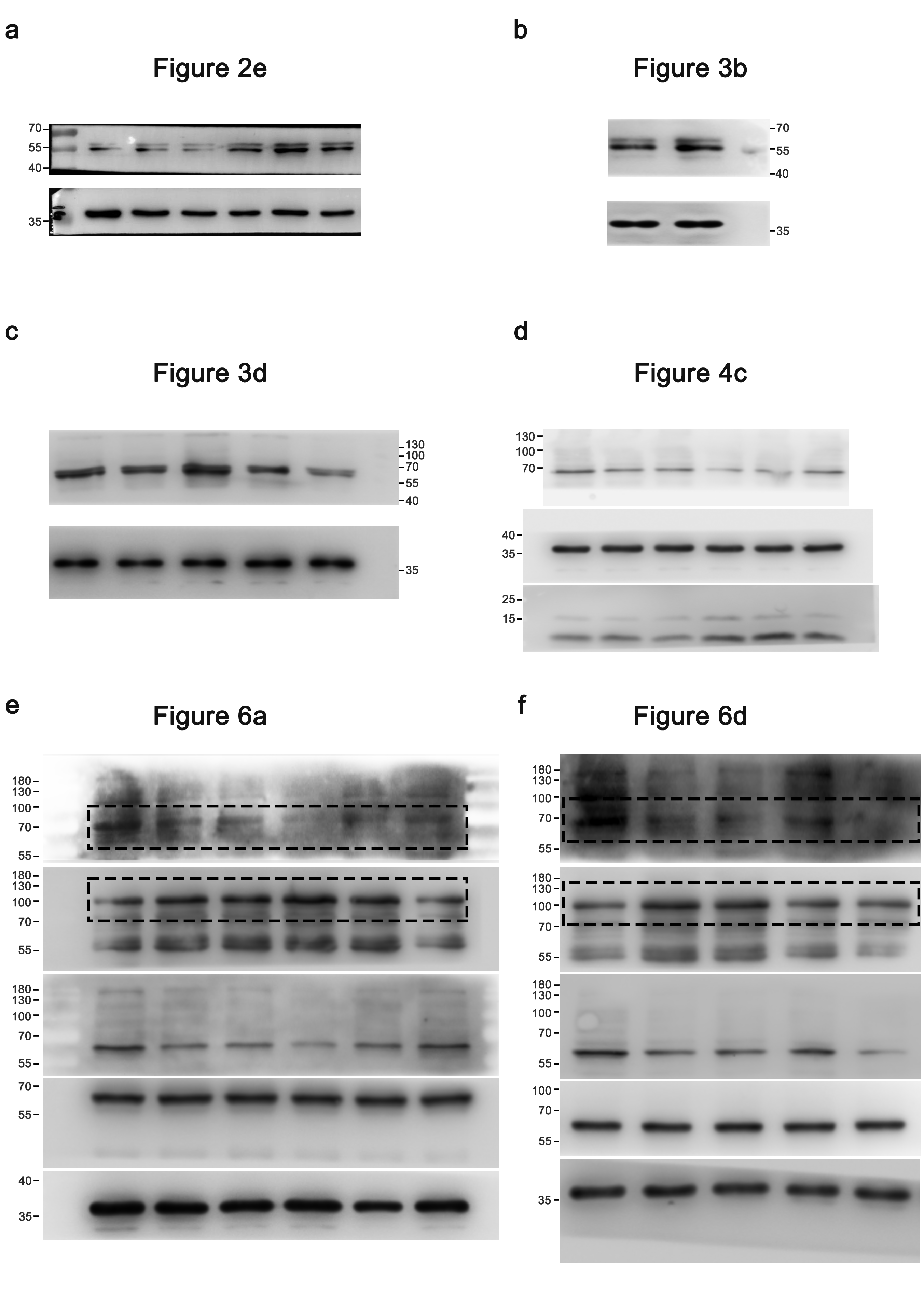

Supplement: FIGURE S2 — Uncropped blot images. [file Image_2.TIF]
